# Pulse Coupled Neural Network-Based Multimodal Medical Image Fusion via Guided Filtering and WSEML in NSCT Domain

**DOI:** 10.3390/e23050591

**Published:** 2021-05-11

**Authors:** Liangliang Li, Hongbing Ma

**Affiliations:** Department of Electronic Engineering, Tsinghua University, Beijing 100084, China; leeliangliang@tsinghua.edu.cn

**Keywords:** multimodal medical image, image fusion, PCNN, WSEML, GIF, NSCT

## Abstract

Multimodal medical image fusion aims to fuse images with complementary multisource information. In this paper, we propose a novel multimodal medical image fusion method using pulse coupled neural network (PCNN) and a weighted sum of eight-neighborhood-based modified Laplacian (WSEML) integrating guided image filtering (GIF) in non-subsampled contourlet transform (NSCT) domain. Firstly, the source images are decomposed by NSCT, several low- and high-frequency sub-bands are generated. Secondly, the PCNN-based fusion rule is used to process the low-frequency components, and the GIF-WSEML fusion model is used to process the high-frequency components. Finally, the fused image is obtained by integrating the fused low- and high-frequency sub-bands. The experimental results demonstrate that the proposed method can achieve better performance in terms of multimodal medical image fusion. The proposed algorithm also has obvious advantages in objective evaluation indexes VIFF, Q_W_, API, SD, EN and time consumption.

## 1. Introduction

**Figure 1:**
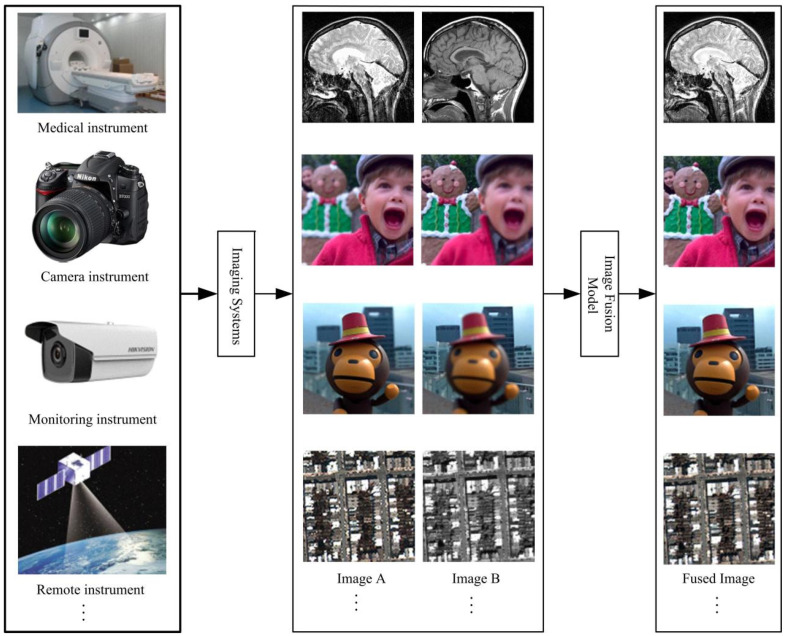
The example of image fusion.

In recent years, numerous medical image processing algorithms are being extensively used for visualizing complementary information. Medical image fusion is a very effective technique in combining the important information obtained from the multimodal images into one single composite image and enhance the diagnostic accuracy [1,2]. Medical images can be divided into the following categories: Computed tomography (CT), magnetic resonance imaging (MRI), positron emission tomography (PET), single-photon emission CT (SPECT) etc. Usually, there is no single imaging method that can reflect the complete tissue information; medical image fusion technology can retain the diagnostic information of input image to the maximum extent [3,4]. Figure 1 shows the example of image fusion, it involves not only medicine, but also a multifocus image and remote sensing image. In this paper, we mainly discuss the application of multimodal medical image fusion.

At present, a lot of image fusion techniques have been proposed by the researchers, and these image fusion methods are broadly categorized as spatial domain and transform domain [5,6]. The spatial domain-based image fusion methods have high computational efficiency, but these methods suffer from poor contrast and spatial localization [7,8]. In terms of technical development, many multiscale transform decomposition methods have been introduced to design an effective platform that provide better localization of an image contour and texture details [9]. These transforms include the discrete wavelet transform (DWT) [10], stationary wavelet transform (SWT) [11], dual-tree complex wavelet transform (DTCWT) [12], curvelet transform (CVT) [13], contourlet transform (CNT) [14], surfacelet transform [15], non-subsampled contourlet transform (NSCT) [16], shearlet transform (ST) [17], non-subsampled shearlet transform (NSST) [18], adjustable non-subsampled shearlet transform (ANSST) [19] etc. Iqbal et al. [20] proposed a novel multifocus image fusion scheme utilizing discrete wavelet transform and guided image filtering, which can provide outperformance fusion results both on qualitative and quantitative comparisons. Wang et al. [21] introduced a technique for multifocus image fusion based on discrete wavelet transform and convolutional neural network (CNN), leading to better fusion results than traditional DWT-based fusion algorithm. DTCWT is an extension of DWT and has translation invariance. Aishwarya et al. [22] proposed an image fusion method utilizing DTCWT and adaptive combined clustered dictionary, leading to good performance than the conventional multiscale transform-based algorithms and the state-of-the-art sparse representation-based algorithms.

Due to the limited ability in capturing directional information in two-dimensional space about the wavelets based methods, most wavelet transforms cannot generate an optimal representation for images. In order to address the aforementioned problem, a series of multi-scale geometric analysis (MGA) theory including curvelet, contourlet and shearlet have been introduced by the scientist, these methods accelerate the development of image fusion technology. Mao et al. [23] proposed an image fusion technique based on curvelet transform and sparse representation. Chen et al. [24] introduced an approach for multi-source optical remote sensing image fusion based on principal component analysis and curvelet transform. Li et al. [25] introduced the non-subsampled contourlet transform into the medical image fusion based on fuzzy entropy and regional energy. Wu et al. [26] conducted another NSCT-based work using pulse coupled neural network (PCNN) for infrared and visible image fusion. Li et al. [27] proposed an image fusion scheme based on parameter-adaptive pulse coupled neural network (PAPCNN) and improved sum-modified-laplacian (ISML) in non-subsampled shearlet transform (NSST) domain, leading to a good fusion performance.

In recent years, the sparse representation-based, convolutional neural network-based, edge-preserving filter-based techniques also achieve successfully in the field of image fusion. Xing et al. [28] proposed an image fusion method based on Taylor expansion theory and convolutional sparse representation with gradient penalties scheme. Liu et al. [29] introduced an adaptive sparse representation (ASR) for multimodal image fusion and denoising. Liu et al. [30] proposed an image fusion technique using deep convolutional neural network (DCNN), leading to state-of-the-art image fusion performance in terms of visual quality and objective assessment. Li et al. [31] introduced the guided image filtering for image fusion (GFF), and the calculation efficiency is relatively high. The main image fusion models mentioned above can be summarized as shown in Table 1.

The image fusion methods, based on transform domain, mainly use different energy functions to construct the weight of the source image for image fusion. Although the details of each source image can be well-preserved, the space continuity of the high- and low-frequency coefficients in the transform domain is often not considered, the fused image will introduce artificial texture, which will affect the image fusion effect. In this paper, a novel fusion model with pulse coupled neural network (PCNN) and weighted sum of eight-neighborhood-based modified Laplacian (WSEML) in NSCT domain is proposed for multimodal medical image fusion. The guided filtering is introduced to enhance the spatial continuity of the image, and then the corresponding artificial texture is suppressed and the gray level of the fused image is enhanced. The contributions of the proposed framework can be summarized as follows: (1) The multiscale NSCT decomposition is used to decompose the input source images into low- and high-frequency components; (2) the PCNN is adopted to fuse the low-frequency components, and the WSEML integrating guided image filtering is utilized to fuse the high-frequency components. The guided image filtering is a good edge-preserving filter, the proposed model can efficiently capture the spatial information and suppress noise; (3) the effectiveness of the proposed work is authenticated utilizing the extensive experimental fusion results and comparisons with the state-of-the-art image fusion algorithms.

The rest of this work is organized as follows. Preliminaries is briefly reviewed in Section 2. The proposed fusion algorithm is illustrated in Section 3. The experimental results and discussions are shown in Section 4. The conclusions are presented in Section 5.

## 2. Preliminaries

### 2.1. Non-Subsampled Contourlet Transform

**Figure 2:**
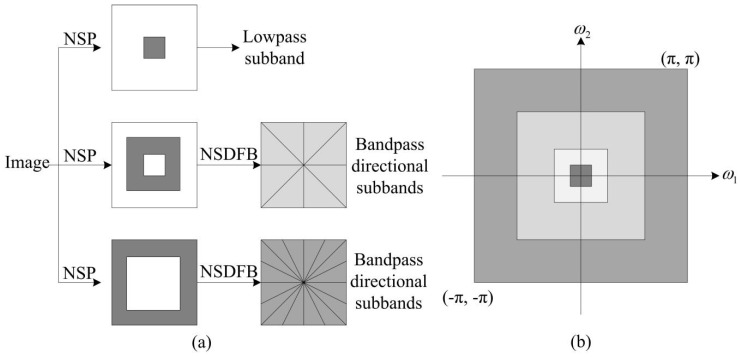
The overview of NSCT [32]. (**a**) Non-subsampled filter bank structure; (**b**) Idealized frequency partitioning.

The non-subsampled contourlet transform (NSCT) is an improved model of contourlet, NSCT adopts the multiscale, multidirectional analysis and shift-invariance. It is applied for image decomposition into one low-frequency and several high-frequency sub-bands. The decomposition model utilizes non-subsampled pyramid (NSP) to generate low-frequency and high-frequency components and then the non-subsampled directional filter bank (NSDFB) is adopted to generate several sub-image components [32]. The overview of NSCT is depicted in Figure 2. NSCT is recognized as an effective method for image fusion [25,26], and it is selected as the multiscale transform for proposed fusion algorithm in this paper.

### 2.2. Pulse Coupled Neural Network

**Figure 3:**
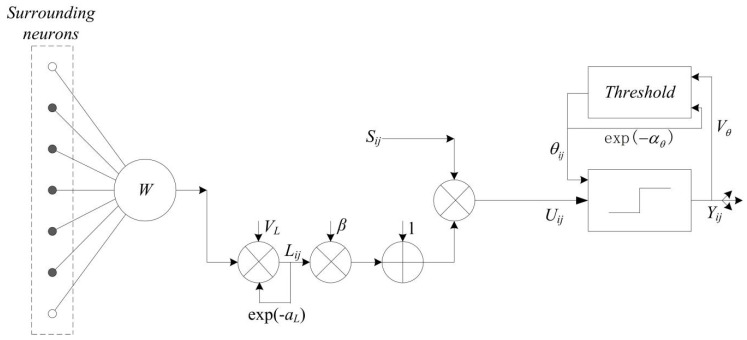
Architecture of the PCNN model.

Pulse coupled neural network (PCNN) is a feedback network, and it is widely used in the field of image fusion. In particular, it is reasonable to apply the PCNN model to merge the low-frequency components generated by the NSCT. The PCNN model is described as follows [16]:

(1) $$F_{ij}\left(n\right)\;=S_{ij}$$

(2) $$L_{ij}\left(n\right)\;=e^{-\alpha_{L}}L_{ij}\left(n-1\right)\;+\;V_{L}\sum_{pq}W_{ij,pq}Y_{ij,pq}\left(n-1\right)$$

(3) $$U_{ij}\left(n\right)\;=F_{ij}\left(n\right)\;*\;\left(1+\beta L_{ij}\left(n\right)\right)$$

(4) $$\theta_{ij}\left(n\right)\;=e^{-\alpha_{\theta }}\theta_{ij}\left(n-1\right)\;+\;V_{\theta }Y_{ij}\left(n-1\right)$$

(5) $$Y_{ij}\left(n\right)\;=\;\left\{\begin{array}{ll} 1 & \mathrm{if}\;U_{ij}\left(n\right)\;>\theta_{ij}\left(n\right)\\ 0 & \mathrm{else} \end{array}\right.$$

(6) $$T_{i,j}=T_{i,j}\left(n-1\right)\;+\;Y_{i,j}\left(n\right)$$

where $F_{ij}$ shows the feeding input and *S_ij_* denotes the external input stimulus signal, the linking input $L_{ij}$ depicts the sum of neurons firing times in linking range, $W_{ij,pq}$ represents the synaptic gain strength, $\alpha_{L}$ denotes the decay constants, $V_{L}$ and $V_{\theta }$ present the amplitude gain, β shows the linking strength, $U_{ij}$ is the total internal activity, $\theta_{ij}$ represents the threshold, n is the iteration times, *Y_ij_* is the pulse output of PCNN, $T_{ij}$ denotes the firing times. Figure 3 shows the architecture of the PCNN model.

### 2.3. Guided Image Filter

Guided image filter is a linear filtering, we suppose that the filtering output image *q* is the linear transform of the guidance image *I* in a window $\omega_{k}$ centered at the pixel k [33]:

(7) $$q_{i}=a_{k}I_{i}+b_{k},\forall i\in \omega_{k}$$

where $\omega_{k}$ presents the square window of size $\left(2r+1\right)\;\times \;\left(2r+1\right)$. The linear coefficients $\left(a_{k},b_{k}\right)$ are constant in the $\omega_{k}$, and they could be estimated by minimizing the cost function in the window $\omega_{k}$:

(8) $$E\left(a_{k},b_{k}\right)\;=\sum_{i\in \omega_{k}}\left({\left(a_{k}I_{i}+b_{k}-p_{i}\right)}^{2}+\varepsilon a_{k}^{2}\right)$$

where ε represents the regularization parameter penalizing large $a_{k}$. The linear coefficients $\left(a_{k},b_{k}\right)$ can be calculated by the following:

(9) $$a_{k}=\frac{\frac{1}{\left|\omega \right|}\sum_{i\in \omega_{k}}I_{i}p_{i}-\mu_{k}{\bar{p}}_{k}}{\sigma_{k}^{2}+\varepsilon }$$

(10) $$b_{k}={\bar{p}}_{k}-a_{k}\mu_{k}$$

where $\mu_{k}$ and $\sigma_{k}^{2}$ denote the mean and variance of *I* in $\omega_{k}$, $\left|\omega \right|$ shows the number of pixels in $\omega_{k}$, and ${\bar{p}}_{k}$ represents the mean of p in $\omega_{k}$, it can be calculated by the following:

(11) $${\bar{p}}_{k}=\frac{1}{\left|\omega \right|}\sum_{i\in \omega_{k}}p_{i}$$

In order to keep the $q_{i}$ value unchanged in different windows, all the possible data of $\left(a_{k},b_{k}\right)$ are first averaged, the filtering output can be computed by

(12) $$q_{i}=\frac{1}{\left|\omega \right|}\sum_{k|i\in \omega_{k}}\left(a_{k}I_{i}+b_{k}\right)\;={\bar{a}}_{i}I_{i}+{\bar{b}}_{i}$$

where ${\bar{a}}_{i}$ and ${\bar{b}}_{i}$ present the mean of $a_{k}$ and $b_{k}$, respectively; they can be computed by

(13) $${\bar{a}}_{i}=\frac{1}{\left|\omega \right|}\sum_{k\in \omega_{i}}a_{k}$$

(14) $${\bar{b}}_{i}=\frac{1}{\left|\omega \right|}\sum_{k\in \omega_{i}}b_{k}$$

In this work, the $G_{r,\varepsilon }\left(p,I\right)$ is utilized to denote the guided filtering operation, r and ε denote the parameters which control the size of filter kernel and blur extent, respectively. p refers to the input image, and I represents the guidance image. The guided image filter is used to process the high-frequency components generated by NSCT.

## 3. Proposed Fusion Method

### 3.1. Overview

**Figure 4:**
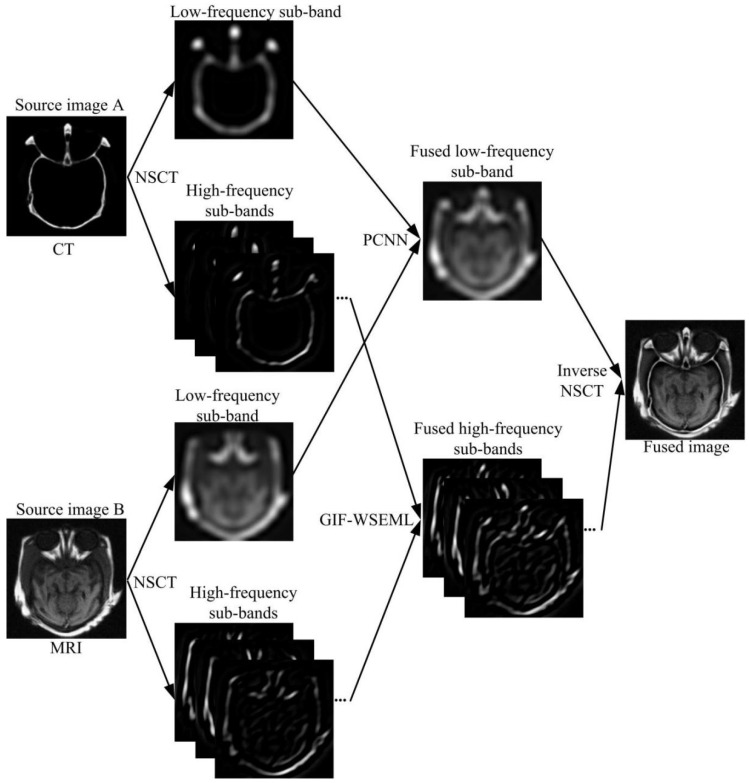
The schematic diagram of the proposed fusion method.

The proposed multimodal medical image fusion algorithm in this work is shown in Figure 4. The input source images are assumed to be well registered with the size 256 × 256, the detailed image fusion approach consists of four parts, namely NSCT decomposition, low-frequency sub-bands fusion, high-frequency sub-bands fusion, and NSCT reconstruction.

### 3.2. Detailed Fusion Algorithm

**Step 1:** NSCT decomposition

Suppose the registered input source images *A* and *B* are decomposed by NSCT transform with *L*-level, and generate the corresponding decomposition low- and high-frequency sub-bands $\left\{L_{A},L_{B}\right\}$ and $\left\{H_{A}^{l,k},H_{B}^{l,k}\right\}$, respectively.

**Step 2:** Low-frequency sub-band fusion

The low-frequency sub-band contains the approximate information of the source images, in this section, the PCNN based fusion rule is applied to keep more useful information. According to the PCNN model described from Equations (1)–(6), the fusion rule is depicted in the following:

(15) $$D_{F}\left(i,j\right)\;=\;\left\{\begin{array}{ll} 1 & if\;T_{A,ij}\left(N\right)\;\geq T_{B,ij}\left(N\right)\\ 0 & else \end{array}\right.$$

(16) $$L_{F}\left(i,j\right)\;=\;\left\{\begin{array}{ll} L_{A}\left(i,j\right) & if\;\;D_{ij}\left(N\right)=1\\ L_{B}\left(i,j\right) & else \end{array}\right.$$

where $T_{A,ij}\left(N\right)$ and $T_{B,ij}\left(N\right)$ are the PCNN firing times, *N* presents the total number of iterations; $D_{ij}$ represents the decision map, $L_{F}\left(i,j\right)$ denotes the fused low-frequency sub-band.

**Step 3:** High-frequency sub-bands fusion

The high-frequency sub-bands contain the plentiful edge and texture detail information of the input images, in order to extract the details information, the weighted sum of eight-neighborhood-based modified Laplacian (WSEML) is adopted, and it is defined as follows [34]:

(17) $${\mathrm{WSEML}}_{S}\left(i,j\right)\;=\sum_{m=-r}^{r}\;\sum_{n=-r}^{r}W\left(m+r+1,n+r+1\right)\;\times \;{\mathrm{EML}}_{S}\left(i+m,j+n\right)$$

(18) $$\begin{array}{cc} {\mathrm{EML}}_{S}\left(i,j\right)\;= & \left|2S\left(i,j\right)-S\left(i-1,j\right)-S\left(i+1,j\right)\right|\\ & +\left|2S\left(i,j\right)-S\left(i,j-1\right)-S\left(i,j+1\right)\right|\\ & +\frac{1}{\sqrt{2}}\left|2S\left(i,j\right)-S\left(i-1,j-1\right)-S\left(i+1,j+1\right)\right|\\ & +\frac{1}{\sqrt{2}}\left|2S\left(i,j\right)-S\left(i-1,j+1\right)-S\left(i+1,j-1\right)\right| \end{array}$$

where $S\in \left\{A,B\right\}$; W denotes the weighting matrix, and it can be calculated by the following:

(19) $$W=\frac{1}{16}\left[\begin{array}{ccc} 1 & 2 & 1\\ 2 & 4 & 2\\ 1 & 2 & 1 \end{array}\right]$$

For the high-frequency coefficients, the fusion rule based on WSEML is adopted, and then the two zero-value matrixes *mapA* and *mapB* are initialized, and the matrixes are computed by the following:

(20) $$mapA\left(i,j\right)\;=\;\left\{\begin{array}{ll} 1 & if\;WSEML_{H_{A}^{l,k}}\left(i,j\right)\;\geq WSEML_{H_{B}^{l,k}}\left(i,j\right)\\ 0 & else \end{array}\right.$$

(21) $$mapB(i,j)=1-mapA\left(i,j\right)$$

In order to enhance the spatial continuity of the high-frequency coefficients, the guided filter on *mapA* and *mapB* is adopted, and the corresponding coefficients $H_{A}^{l,k}$ and $H_{B}^{l,k}$ are utilized as the guided images:

(22) $$mapA=G_{r,\varepsilon }\left(mapA,H_{A}^{l,k}\right)$$

(23) $$mapB=G_{r,\varepsilon }\left(mapB,H_{B}^{l,k}\right)$$

where *mapA* and *mapB* should be normalized, the fused high-frequency coefficients $H_{F}^{l,k}\left(i,j\right)$ can be generated by the following Equation:

(24) $$H_{F}^{l,k}\left(i,j\right)\;=mapA\times H_{A}^{l,k}\left(i,j\right)\;+\;mapB\times H_{B}^{l,k}\left(i,j\right)$$

**Step 4:** NSCT reconstruction

The final fused image is generated by performing inverse NSCT transform over the merged fusion sub-bands $\left\{L_{F},H_{F}^{l,k}\right\}$.

### 3.3. Extension to Color Image Fusion

**Figure 5:**
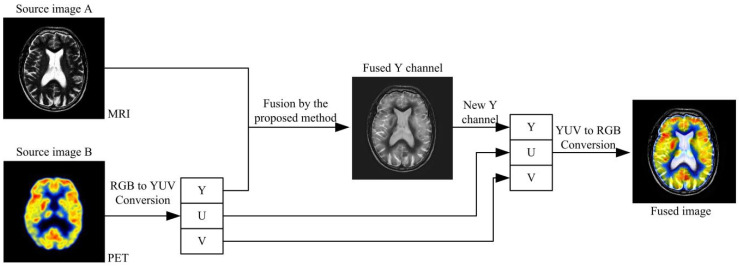
Process flow for the proposed algorithm for anatomical and functional image in YUV color space.

The proposed medical image fusion algorithm is extended to fuse the anatomical and functional image in this section. The anatomical image contains the CT and MRI, and the functional image usually denotes the PET and SPECT. When solving the gray image and color image fusion, the image color space conversion is adopted, in this paper, the RGB to YUV color space is used to compute the anatomical and functional image fusion work [34]. The framework of the anatomical and functional image fusion is shown in Figure 5.

## 4. Experimental Results and Discussions

### 4.1. Experimental Setup

**Figure 6:**
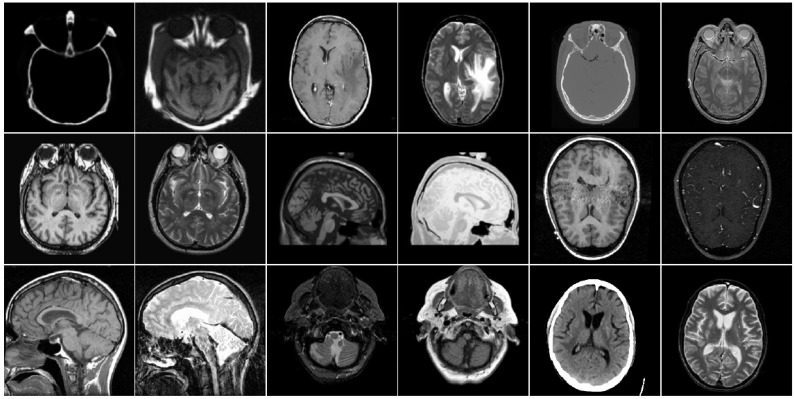
Test gray medical images.

**Figure 7:**
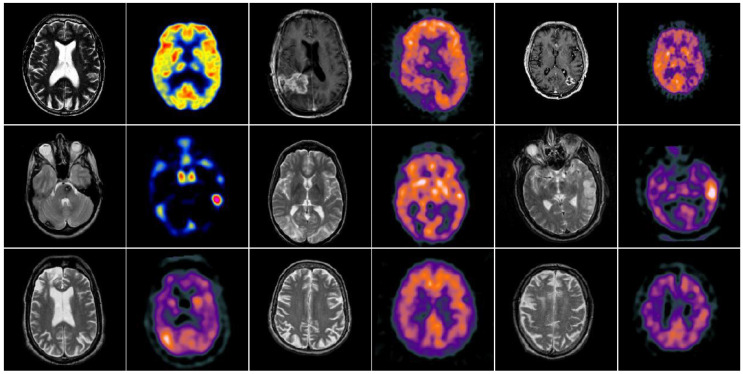
Test anatomical and functional images.

In this section, to explore the effectiveness of the proposed multimodal medical image fusion algorithm, we evaluate the method on the two public datasets http://www.imagefusion.org and http://www.med.harvard.edu/AANLIB/home.html (accessed on 10 February 2021). Figure 6 shows the selected public gray source image fusion pairs, all the CT and MRI source images have the same size with 256 × 256. Figure 7 denotes the selected anatomical and functional (MRI-PET/SPECT) images with the size 256 × 256, and all the source images are pre-registered. In addition, eight state-of-the-art fusion approaches are used to compare with the proposed scheme, namely image fusion based on non-subsampled contourlet transform (NSCT) [16], image fusion using dual-tree complex wavelet transform (DTCWT) [12], guided image filtering for image fusion (GFF) [31], image fusion utilizing ratio of low-pass pyramid (RP) [13], image fusion via adaptive sparse representation (ASR) [29], deep convolutional neural network based image fusion (DCNN) [30], image fusion using convolutional sparsity based morphological component analysis (CSMCA) [35], single-scale structural image decomposition (SSID) [36]. In this paper, the pyramid filter and directional filter with the parameters “9–7” and “pkva"; the NSCT decomposition level is 4, and the corresponding directions are 4, 4, 4, 4; the parameters of the PCNN is set as p×q, $\alpha_{L}=0.06931$, $\alpha_{\theta }=0.2$, β=3, $V_{L}=1.0$, $V_{\theta }=20$, $W=\left[\begin{array}{ccc} 0.707 & 1 & 0.707\\ 1 & 0 & 1\\ 0.707 & 1 & 0.707 \end{array}\right]$, and the iterative number is N=200; the parameters r and ε of guided image filer are set as 3 and 1, respectively. For the parameters in the comparison algorithms, corresponding parameter values are as described in the original papers proposed by the scholars. Table 2 summarizes the tested algorithms and the parameter setup. All of the experiments run in win7, MATLAB R2018b software. The hardware is Intel(R) Core(TM) i5-2520M CUP (2.50 GHz) and 12-GB memory.

**Figure 8:**
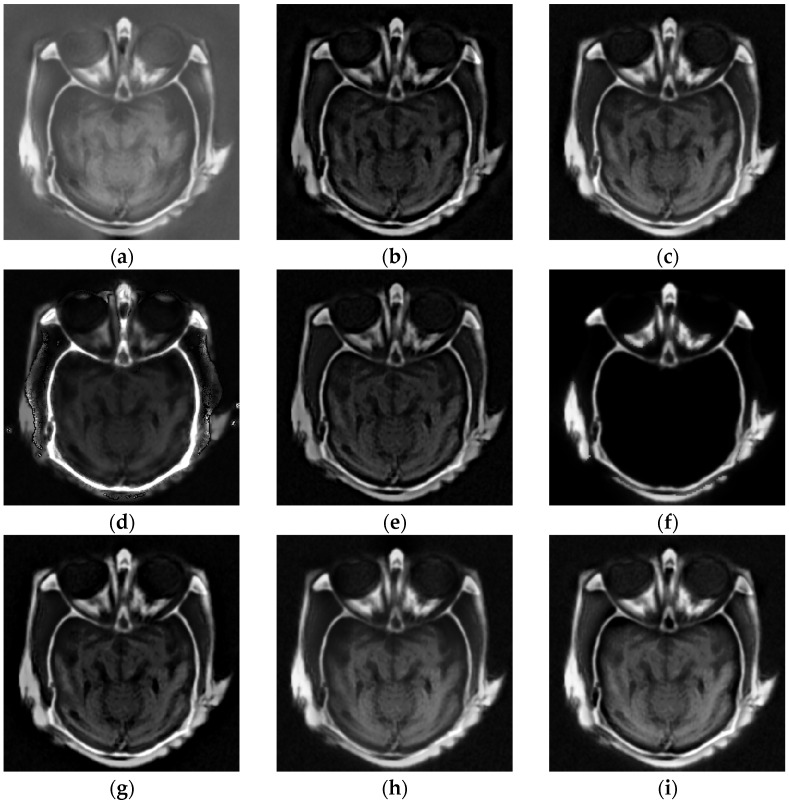
Fusion results of the first group gray medical images. (**a**) NSCT; (**b**) DTCWT; (**c**) GFF; (**d**) RP; (**e**) ASR; (**f**) DCNN; (**g**) CSMCA; (**h**) SSID; (**i**) Proposed method.

**Figure 9:**
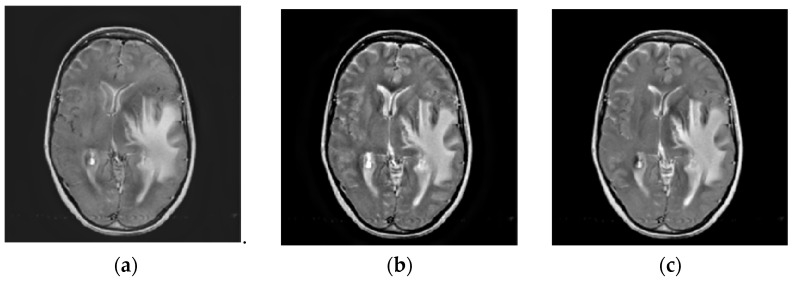

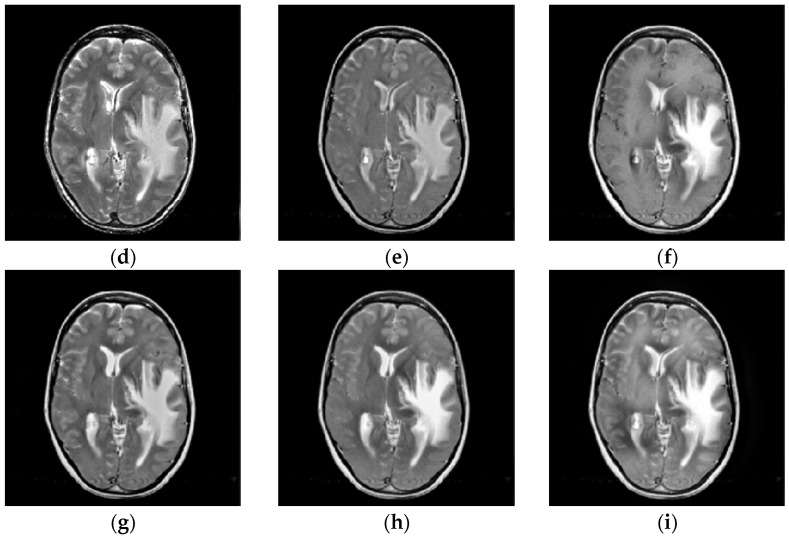
Fusion results of the second group gray medical images. (**a**) NSCT; (**b**) DTCWT; (**c**) GFF; (**d**) RP; (**e**) ASR; (**f**) DCNN; (**g**) CSMCA; (**h**) SSID; (**i**) Proposed method.

**Figure 10:**
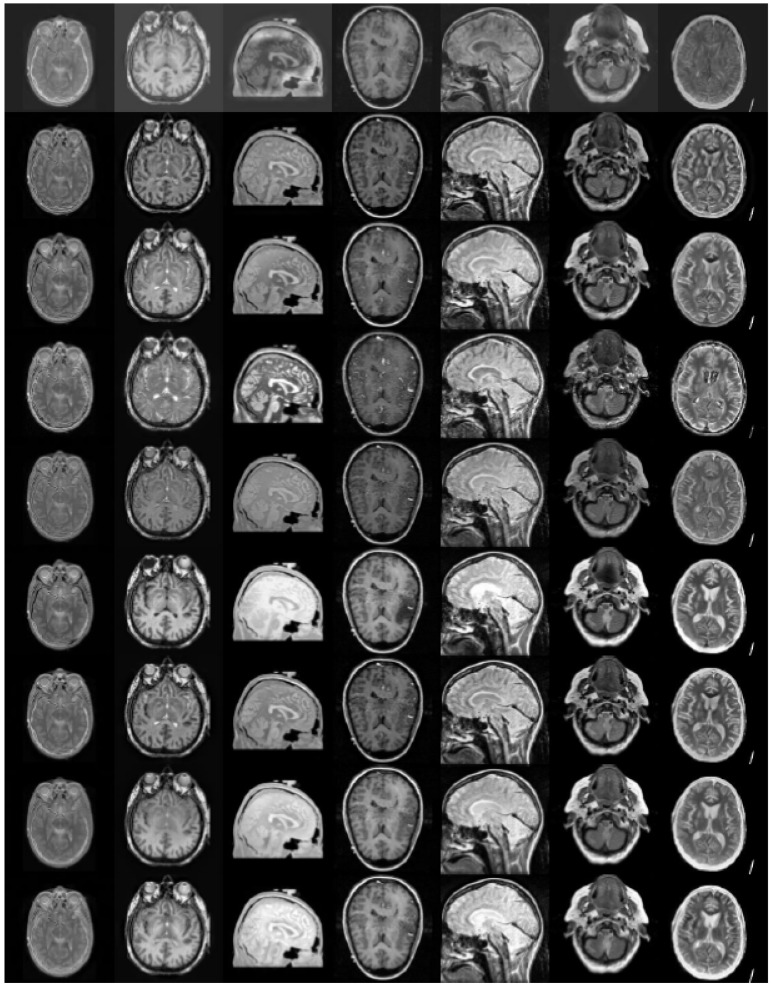
Simulation results of other seven groups of gray medical images in Figure 6. From top to bottom, the fusion results of NSCT, DTCWT, GFF, RP, ASR, DCNN, CSMCA, SSID and proposed method are in turn.

**Figure 11:**
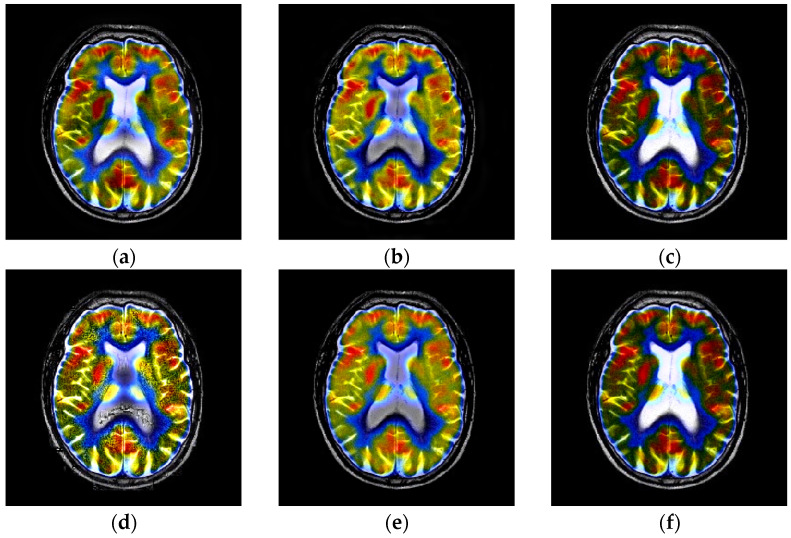

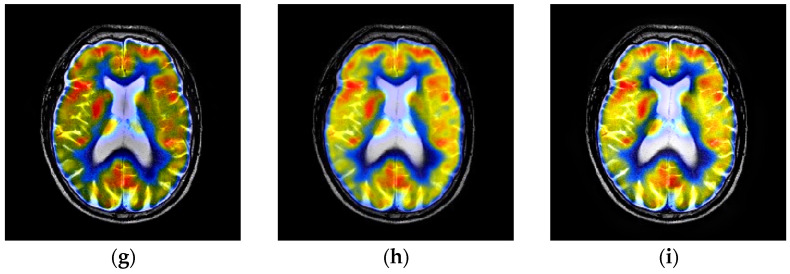
Fusion results of the first group anatomical and functional images. (**a**) NSCT; (**b**) DTCWT; (**c**) GFF; (**d**) RP; (**e**) ASR; (**f**) DCNN; (**g**) CSMCA; (**h**) SSID; (**i**) Proposed method.

**Figure 12:**
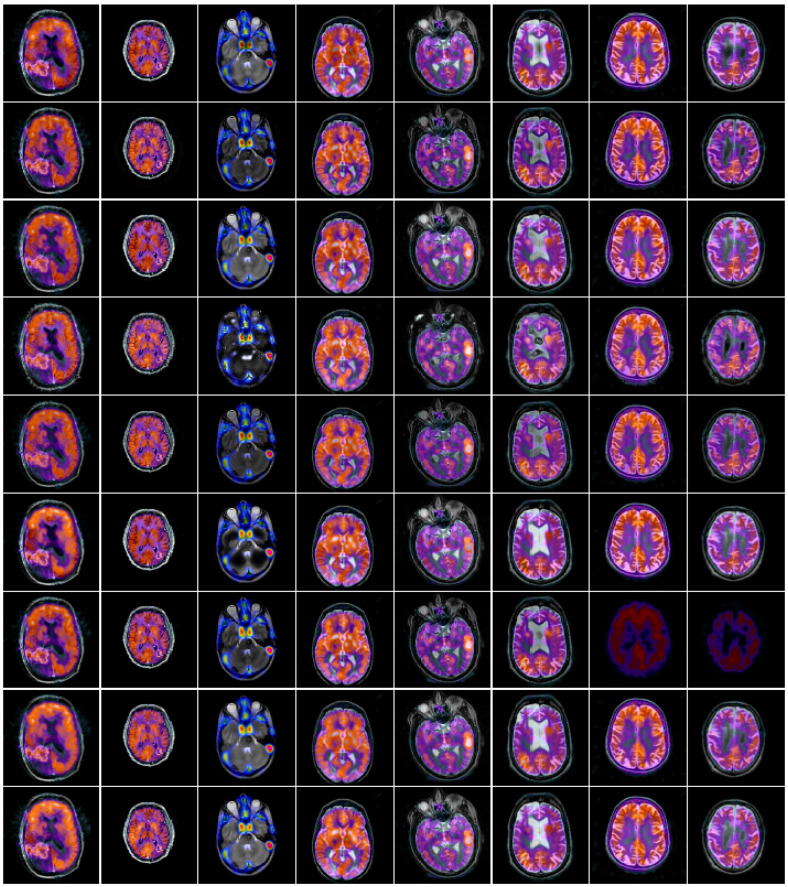
Simulation results of other eight groups of anatomical and functional images in Figure 7. From top to bottom, the fusion results of NSCT, DTCWT, GFF, RP, ASR, DCNN, CSMCA, SSID and proposed method are in turn.

The proposed medical image fusion technique is evaluated and compared with other classical fusion algorithms by qualitative and quantitative analyses. In terms of qualitative analysis, it is based on human visual system such as image details, image contrast and image brightness etc. As for quantitative analysis, multiple evaluation metrics are selected to assess the proposed fusion algorithm and the comparison fusion algorithms, which include the visual information fidelity (VIFF) [37,38,39,40,41], weighted fusion quality index (Q_W_) [42,43], average pixel intensity (API) [44], standard deviation (SD) [44], entropy (EN) [44,45,46,47,48] and time (seconds). VIFF measures the visual information fidelity of the fused image by computing the distortion of the images, a larger VIFF means the fused image has higher visual information fidelity; the Q_W_ addresses the distortions of coefficient correlation, illumination and contrast between the source images and fused image, a larger Q_W_ means less distortion of image quality; API measures an index of contrast, a larger API reflects the fused image has higher contrast; SD measures the amount of information contained in the fused image from the perspective of statistics and reflects the overall contrast, a larger SD reflects the fused image contains more information and higher contrast; the computation of EN value is based on information theory, and it measures the amount of information in the fusion image, a larger EN means the fused image contains more information. The low computation time shows that the algorithm is efficient. Among the examined quantitative metrics, VIFF and Q_W_ are reference-based metrics, while API, SD and EN are no-reference evaluation metrics. The fusion method takes the anatomical or functional image as the reference, it is easy to introduce the interference information from the source images into the fusion image. In order to comprehensively evaluate the fusion performance from different perspectives, this study uses reference-based and no-reference-based indicators. The corresponding fusion results and metrics data as shown in Figure 8, Figure 9, Figure 10, Figure 11 and Figure 12 and Table 3, Table 4, Table 5, Table 6 and Table 7.

### 4.2. Comparison of Gray Image Fusion

Figure 8, Figure 9 and Figure 10 represent the gray medical image fusion results generated by different image fusion approaches. Figure 8 depicts the fused results of the methods on the first group gray medical images. Figure 9 presents the fusion results of the algorithms on the second group gray medical images. Figure 10 shows the fused results of the methods on other gray medical images.

With regard to the visual performance, the edge information in Subfigure (a) of Figure 8 and Figure 9 denotes that the fused images of NSCT have lost some details of MRI images, and the results have some noise, which affects the doctor’s observation. From the Subfigure (b) of Figure 8 and Figure 9 generated by the DTCWT method have the low contrast and brightness. We can denote the blocking artifacts are generated by GFF algorithm as shown in Subfigure (c) of Figure 8 and Figure 9, due to the guided image filtering is a non-linear filter, it needs the same or better guidance image to implement the smoothing process. The fused images calculated by RP and DCNN schemes as shown in Subfigures (d) and (f) of Figure 8 and Figure 9, respectively, and the results produce certain kinds of distortions, especially the Figure 8f obtained by DCNN, almost all the information of MRI image is lost in the fusion image. ASR algorithm can generate the block effect and the gradient contrast is poor, which could be denoted from Subfigure (e) of Figure 8 and Figure 9. It can be seen from Subfigure (g) of Figure 8 and Figure 9 that the fused results computed by CSMCA approach lead to information loss. The fusion results calculated by the SSID and proposed techniques are relatively high-quality, and they are depicted in Subfigures (h) and (i) of Figure 8 and Figure 9, the results of the proposed method retain more image information and the brightness is higher.

In order to reduce the influence of individual subjective judgment on image fusion quality as far as possible, the objective evaluation indicators are introduced, and the corresponding index values are shown in Table 3, Table 4 and Table 5. From the Table 3, in terms of Q_W_, API, SD and EN, the proposed approach generates superb performance, although the best data for VIFF and Time are generated by GFF and SSID, with 0.4863 and 0.1608, respectively. From the Table 4, we can see that the values of VIFF, Q_W_, API and SD obtained by the proposed fusion scheme are the best, while the best data for EN and Time are generated by GFF and SSID, with 5.3836 and 0.0721, respectively. In order to analyze the universality of the fusion algorithms more objectively, we take the average values of the index data obtained from nine groups of gray medical images computed by the nine fusion methods, as shown in Table 5, in addition to the EN and Time values, the other four metric values obtained by the proposed algorithm are the best.

### 4.3. Comparison of Anatomical and Functional Image Fusion

In this section, nine groups of color medical images (MRI-PET/SPECT) are used to assess the fused results of the proposed fusion technique, and the corresponding comparative analysis is given. The typical MRI-PET fusion results of the techniques are given in Figure 11. From the Figure 11, we can denote that the fused images such as Figure 11a–c,f generated by the NSCT, DTCWT, GFF, and DCNN algorithms, respectively, suffer from color distortion. Figure 11d–e are the fusion results computed by the RP and ASR methods, respectively; it can be clearly denoted that the fused results still exists the color distortion, but the image contrast and brightness have improved. The fused image computed by the CSMCA is shown in Figure 11g, and the artificial textures are appeared, the fusion effects are undesirable. The fused images calculated by SSID and the proposed methods are depicted in Figure 11h,i, respectively, the two fused images are similar, but the proposed method has a better fusion performance and higher brightness. Figure 12 shows the fused results of different algorithms on the other eight groups of anatomical and functional images.

The quantitative assessments on the fused images of Figure 11 corresponding to the first group anatomical and functional images are tabulated in Table 6. We can see that the metrics data of API, SD and EN computed by the proposed algorithm are the best compared with other state-of-the-art fusion strategies, while the best data for VIFF, Q_W_ and Time are computed by RP and SSID, with 0.8443, 0.8471and 0.0806, respectively.

Here, the average of the six metrics calculated by the various fusion approaches on the selected nine groups anatomical and functional images in Figure 7 are recorded, as shown in Table 7. In contrast to the other fusion techniques, there is a remarkable enhancement on the metrics API, SD and EN. The overall comparative analysis shows that the proposed scheme works better in terms of anatomical and functional images fusion, demonstrating its effectiveness.

From the anatomical-anatomical image fusion results and anatomical-functional image fusion results aforementioned, the proposed algorithm has obvious advantages in subjective and objective evaluations compared with other state-of-the-art fusion algorithms. The PCNN fusion rule and GIF-WSEML fusion rule are used in the NSCT domain, the combination of the two fusion models denotes better preservation of spatial and spectral features. The fusion images can provide accurate location of defected tissues, and provide meaningful quantitative explanation for clinical diagnosis. Given that there are many parameters in this algorithm, it needs continuous manual debugging to select the appropriate values of parameters to achieve the optimal fusion effect.

## 5. Conclusions

In this paper, a practical multimodal medical image fusion algorithm based on PCNN and GIF-WSEML in non-subsampled contourlet transform domain is introduced. For sub-bands fusion, two different rules are adopted, the low-frequency sub-bands are fused by PCNN model, and the weighted sum of eight-neighborhood-based modified Laplacian integrating guided image filtering (GIF-WSEML) is used to merge the high-frequency sub-bands. The nine groups of anatomical-anatomical images and nine groups of anatomical-functional images are used to simulate by the proposed framework and other conventional fusion approaches. The comparative experimental fusion results conducted on both gray and color medical image datasets demonstrate that the proposed fusion algorithm has a better performance with improved brightness and contrast of multimodal medical images, and the objective metrics such as VIFF, Q_W_, API, SD and EN computed by the proposed method also have obvious advantages. Compared to DTCWT, GFF, RP and SSID, the time consuming of the proposed method is high, so reducing the operation time and improving the real-time performance of the algorithm are the problems we need to solve in the future.

## Figures and Tables

**Table 1:** The classifications and methods of main image fusion models.

Categories	Methods
Multiscale transform decomposition	discrete wavelet transform (DWT) [10], stationary wavelet transform (SWT) [11], dual-tree complex wavelet transform (DTCWT) [12], curvelet transform (CVT) [13], contourlet transform (CNT) [14],
	surfacelet transform [15], non-subsampled contourlet transform (NSCT) [16], shearlet transform (ST) [17], nonsubsampled shearlet transform (NSST) [18], adjustable non-subsampled shearlet transform (ANSST) [19]
Sparse representation	convolutional sparse representation [28],
	adaptive sparse representation (ASR) [29]
Deep learning	deep convolutional neural network (DCNN) [30]
Edge-preserving filter	guided image filtering [31]

**Table 2:** All tested algorithms and the parameter settings.

Methods	Parameter Setting
NSCT [16]	PCNN is set as p×q, $\alpha_{L}=0.06931$, $\alpha_{\theta }=0.2$, β=0.2, $V_{L}=1.0$, $V_{\theta }=20$, $W=\left[\begin{array}{ccc} 0.707 & 1 & 0.707\\ 1 & 0 & 1\\ 0.707 & 1 & 0.707 \end{array}\right]$, and N=200; the NSCT decomposition direction numbers are [4, 4, 4, 4].
DTCWT [12]	*L* = 4
GFF [31]	$r_{1}=45,\;\varepsilon_{1}=0.3,\;r_{2}=7,\;\varepsilon_{2}=10^{-6}$
RP [13]	*L* = 4
ASR [29]	dictionary size: 256, ε=0.1, C=1.15, σ=0, the number of sub-dictionaries: 7
DCNN [30]	patch size = 16 × 16, convolutional layer: kernel size = 3 × 3, stride = 1, max-pooling layer: kernel size = 2 × 2, stride = 2
CSMCA [35]	$L=6,\;\lambda_{c}=\lambda_{t}=\max\left(0.6-0.1\times i,\;0.005\right),\;i\in [1,\;L]$
SSID [36]	r = 15
Proposed	PCNN is set as p×q, $\alpha_{L}=0.06931$, $\alpha_{\theta }=0.2$, β=3, $V_{L}=1.0$, $V_{\theta }=20$, $W=\left[\begin{array}{ccc} 0.707 & 1 & 0.707\\ 1 & 0 & 1\\ 0.707 & 1 & 0.707 \end{array}\right]$, and N=200; the NSCT decomposition direction numbers are [4, 4, 4, 4], r=3, ε=1

Notes: NSCT (non-subsampled contourlet transform), DTCWT (dual-tree complex wavelet transform), GFF (guided image filtering for image fusion), RP (ratio of low-pass pyramid), ASR (adaptive sparse representation), DCNN (deep convolutional neural network), CSMCA (convolutional sparsity based morphological component analysis), SSID (single-scale structural image decomposition).

**Table 3:** Objective assessment of different fusion methods on the first group gray images.

	VIFF	Q_W_	API	SD	EN	Time/s
NSCT	0.3440	0.7833	40.3719	49.9211	6.6284	23.3362
DTCWT	0.3747	0.7481	32.5113	42.9503	6.2258	0.2269
GFF	0.4863	0.8337	50.1930	53.7113	6.7920	0.2579
RP	0.2256	0.5289	36.4669	51.5819	6.0500	0.2034
ASR	0.3744	0.7526	31.5150	40.0483	6.1778	91.1108
DCNN	0.2398	0.6949	22.3834	52.2447	3.4737	75.3303
CSMCA	0.4752	0.8030	37.2620	50.7438	6.3268	200.6023
SSID	0.4423	0.7988	51.2897	52.4270	6.6580	0.1608
Proposed	0.4594	0.8438	53.2905	55.1511	6.8000	17.9221

**Table 4:** Objective assessment of different fusion methods on the second group gray images.

	VIFF	Q_W_	API	SD	EN	Time/s
NSCT	0.4728	0.8324	56.2619	69.6178	5.2291	22.4744
DTCWT	0.4830	0.8326	52.1862	65.5521	4.9310	0.1799
GFF	0.4850	0.8448	54.5311	65.9081	5.3836	0.2404
RP	0.3582	0.5464	55.5456	70.0442	4.5744	0.1278
ASR	0.4680	0.8164	51.5346	63.9370	4.1560	87.0228
DCNN	0.4638	0.8279	60.4476	74.8379	4.5250	78.8741
CSMCA	0.4940	0.8444	53.2322	67.4899	4.3896	205.1055
SSID	0.5122	0.8426	55.8888	70.3751	4.5738	0.0721
Proposed	0.5151	0.8492	60.6443	75.1231	5.0524	18.5094

**Table 5:** Average objective assessment of different fusion methods on the nine groups gray medical images in Figure 6.

	VIFF	Q_W_	API	SD	EN	Time/s
NSCT	0.5210	0.7761	59.8996	65.1086	6.1218	23.7192
DTCWT	0.5181	0.7713	54.4182	59.9131	5.7897	0.1778
GFF	0.5095	0.7813	60.0666	62.8036	6.0636	0.2568
RP	0.3701	0.5758	58.8046	64.2301	5.6415	0.1428
ASR	0.4824	0.7584	53.6929	57.2958	5.3715	106.4758
DCNN	0.5439	0.7674	65.3528	73.7230	5.1390	80.0550
CSMCA	0.5473	0.7822	56.8599	63.2075	5.4745	199.1734
SSID	0.5970	0.7934	66.2517	70.0540	5.6540	0.0848
Proposed	0.6121	0.8072	70.5363	74.2915	5.9685	19.0577

**Table 6:** Objective assessment of different fusion methods on the first group anatomical and functional images.

	VIFF	Q_W_	API	SD	EN	Time/s
NSCT	0.2651	0.7986	43.1364	64.8996	4.7648	28.2017
DTCWT	0.5901	0.8250	43.4533	62.9923	4.6493	0.1937
GFF	0.1899	0.8075	33.8746	64.0359	4.4073	0.2377
RP	0.8443	0.8471	45.8674	68.7058	4.7289	0.1570
ASR	0.3150	0.7602	42.9496	61.1235	4.1997	85.6910
DCNN	0.2016	0.8049	36.4412	63.0764	4.5451	80.3691
CSMCA	0.3088	0.7926	44.4419	63.9466	4.5383	193.1375
SSID	0.3675	0.6837	53.5451	74.4686	4.6702	0.0806
Proposed	0.3905	0.7737	57.7310	80.6245	4.9169	20.5294

**Table 7:** Average objective assessment of different fusion methods on the nine groups anatomical and functional images in Figure 7.

	VIFF	Q_W_	API	SD	EN	Time/s
NSCT	0.5016	0.8946	39.8883	56.0495	4.7101	26.5087
DTCWT	0.7396	0.9034	35.7573	50.1217	4.7462	0.2026
GFF	0.4947	0.8995	38.8141	55.1386	4.6584	0.2475
RP	0.6223	0.7878	38.4400	53.6370	4.6522	0.1562
ASR	0.4688	0.8342	35.2421	48.2889	4.3736	92.3286
DCNN	0.4952	0.8936	39.6507	56.7982	4.6641	79.5171
CSMCA	0.3801	0.6798	29.8909	42.2079	4.1895	186.4474
SSID	0.5425	0.8690	41.1085	56.0659	4.6606	0.0828
Proposed	0.5484	0.8968	43.7113	59.6273	4.8847	19.3064

## Data Availability

Not applicable.

## Abbreviations

PCNN	pulse coupled neural network
WSEML	weighted sum of eight-neighborhood-based modified Laplacian
GIF	guided image filtering
NSCT	nonsubsampled contourlet transform
CT	computed tomography
MRI	magnetic resonance imaging
PET	positron emission tomography
SPECT	single-photon emission CT
DWT	discrete wavelet transform
SWT	stationary wavelet transform
DTCWT	dual-tree complex wavelet transform
CVT	curvelet transform
CNT	contourlet transform
ST	shearlet transform
NSST	nonsubsampled shearlet transform
ANSST	adjustable nonsubsampled shearlet transform
CNN	convolutional neural network
MGA	multi-scale geometric analysis
PAPCNN	parameter-adaptive pulse coupled neural network
ISML	improved sum-modified-laplacian
DCNN	deep convolutional neural network
GFF	guided image filtering for image fusion
NSP	nonsubsampled pyramid
NSDFB	nonsubsampled directional filter bank
RP	ratio of low-pass pyramid
ASR	adaptive sparse representation
CSMCA	convolutional sparsity based morphological component analysis
SSID	single-scale structural image decomposition
VIFF	visual information fidelity
Q_W_	weighted fusion quality index
API	average pixel intensity
SD	standard deviation
EN	entropy

## References

[1] Singh S., Anand R.S. (2020). Multimodal medical image sensor fusion model using sparse K-SVD dictionary learning in nonsubsampled shearlet domain. IEEE Trans. Instrum. Meas..

[2] Kong W., Miao Q., Lei Y. (2019). Multimodal sensor medical image fusion based on local difference in non-subsampled domain. IEEE Trans. Instrum. Meas..

[3] Wang Z., Cui Z., Zhu Y. (2020). Multi-modal medical image fusion by Laplacian pyramid and adaptive sparse representation. Comput. Biol. Med..

[4] Zhang L., Zeng G., Wei J. (2020). Multi-modality image fusion in adaptive-parameters SPCNN based on inherent characteristics of image. IEEE Sens. J..

[5] Liu Y., Zhou D., Nie R. (2020). Robust spiking cortical model and total-variational decomposition for multimodal medical image fusion. Biomed. Signal Process. Control.

[6] Zhang Y., Liu Y., Sun P. (2020). IFCNN: A general image fusion framework based on convolutional neural network. Inf. Fusion.

[7] Ma J., Liang P., Yu W. (2020). Infrared and visible image fusion via detail preserving adversarial learning. Inf. Fusion.

[8] Liu Y., Wang L., Cheng J. (2020). Multi-focus image fusion: A survey of the state of the art. Inf. Fusion.

[9] Liu Y., Chen X., Wang Z. (2018). Deep learning for pixel-level image fusion: Recent advances and future prospects. Inf. Fusion.

[10] Talal T., Attiya G. (2020). Satellite image fusion based on modified central force optimization. Multimed. Tools Appl..

[11] Liu S., Chen J., Rahardja S. (2020). A new multi-focus image fusion algorithm and its efficient implementation. IEEE Trans. Circuits Syst. Video Technol..

[12] Singh R., Srivastava R. (2012). Multimodal medical image fusion in dual tree complex wavelet transform domain using maximum and average fusion rules. J. Med. Imaging Health Inform..

[13] Liu Y., Liu S., Wang Z. (2015). A general framework for image fusion based on multi-scale transform and sparse representation. Inf. Fusion.

[14] Do M.N., Vetterli M. (2005). The contourlet transform: An efficient directional multiresolution image representation. IEEE Trans. Image Process..

[15] Li B., Peng H. (2020). Multi-focus image fusion based on dynamic threshold neural P systems and surfacelet transform. Knowl. Based Syst..

[16] Qu X., Yan J., Xiao H. (2008). Image fusion algorithm based on spatial frequency-motivated pulse coupled neural networks in nonsubsampled contourlet transform domain. Acta Autom. Sin..

[17] Guo K., Labate D. (2007). Optimally sparse multidimensional representation using shearlets. Siam J. Math. Anal..

[18] Li L., Wang L., Wang Z. (2019). A novel medical image fusion approach based on nonsubsampled shearlet transform. J. Med. Imaging Health Inform..

[19] Vishwakarma A., Bhuyan M.K. (2019). Image fusion using adjustable non-subsampled shearlet transform. IEEE Trans. Instrum. Meas..

[20] Iqbal M., Riaz M. (2020). A multifocus image fusion using highlevel DWT components and guided filter. Multimed. Tools Appl..

[21] Wang Z., Li X., Duan H. (2019). Multifocus image fusion using convolutional neural networks in the discrete wavelet transform domain. Multimed. Tools Appl..

[22] Aishwarya N., Bennila T.C. (2018). Visible and Infrared image fusion using DTCWT and adaptive combined clustered dictionary. Infrared Phys. Technol..

[23] Mao Q., Zhu Y., Lv C. (2020). Image fusion based on multiscale transform and sparse representation to enhance terahertz images. Opt. Express.

[24] Chen C., He X., Guo B. (2020). A pixel-level fusion method for multi-source optical remote sensing image combining the principal component analysis and curvelet transform. Earth Sci. Inform..

[25] Li W., Lin Q., Wang K. (2020). Improving medical image fusion method using fuzzy entropy and nonsubsampling contourlet transform. Int. J. Imaging Syst. Technol..

[26] Wu C., Chen L. (2020). Infrared and visible image fusion method of dual NSCT and PCNN. PLoS ONE.

[27] Li L., Si Y., Wang L. (2020). A novel approach for multi-focus image fusion based on SF-PAPCNN and ISML in NSST domain. Multimed. Tools Appl..

[28] Xing C., Wang M., Dong C. (2020). Using taylor expansion and convolutional sparse representation for image fusion. Neurocomputing.

[29] Liu Y., Wang Z. (2015). Simultaneous image fusion and denoising with adaptive sparse representation. IET Image Process..

[30] Liu Y., Chen X., Peng H. (2017). Multi-focus image fusion with a deep convolutional neural network. Inf. Fusion.

[31] Li S., Kang X., Hu J. (2013). Image fusion with guided filtering. IEEE Trans. Image Process..

[32] Da A., Zhou J., Do M. (2006). The nonsubsampled contourlet transform: Theory, design, and applications. IEEE Trans. Image Process..

[33] He K., Sun J., Tang X. (2013). Guided image filtering. IEEE Trans. Pattern Anal. Mach. Intell..

[34] Yin M., Liu X., Liu Y. (2019). Medical image fusion with parameter-adaptive pulse coupled neural network in nonsubsampled shearlet transform domain. IEEE Trans. Instrum. Meas..

[35] Liu Y., Chen X., Ward R. (2019). Medical image fusion via convolutional sparsity based morphological component analysis. IEEE Signal Process. Lett..

[36] Li H., Qi X., Xie W. (2020). Fast infrared and visible image fusion with structural decomposition. Knowl. Based Syst..

[37] Han Y., Cai Y., Cao Y. (2013). A new image fusion performance metric based on visual information fidelity. Inf. Fusion.

[38] Li L., Ma H., Jia Z. (2021). A novel multiscale transform decomposition based multi-focus image fusion framework. Multimed. Tools Appl..

[39] Li L., Si Y. (2019). Enhancement of hyperspectral remote sensing images based on improved fuzzy contrast in nonsubsampled shearlet transform domain. Multimed. Tools Appl..

[40] Zhang H., Le Z., Shao Z., Xu H., Ma J. (2021). MFF-GAN: An unsupervised generative adversarial network with adaptive and gradient joint constraints for multi-focus image fusion. Inf. Fusion.

[41] Li L., Si Y. (2020). Brain image enhancement approach based on singular value decomposition in nonsubsampled shearlet transform domain. J. Med. Imaging Health Inform..

[42] Liu Z., Blasch E. (2012). Objective assessment of multiresolution image fusion algorithms for context enhancement in night vision: A comparative study. IEEE Trans. Pattern Anal. Mach. Intell..

[43] Wang L., Li B., Tian L. (2014). EGGDD: An explicit dependency model for multi-modal medical image fusion in shift-invariant shearlet transform domain. Inf. Fusion.

[44] Kumar B.K.S. (2015). Image fusion based on pixel significance using cross bilateral filter. Signal Image Video Process..

[45] Du J., Li W. (2020). Two-scale image decomposition based image fusion using structure tensor. Int. J. Imaging Syst. Technol..

[46] Ma J., Zhou Y. (2020). Infrared and visible image fusion via gradientlet filter. Comput. Vis. Image Underst..

[47] Xu H., Ma J., Zhang X. (2020). MEF-GAN: Multi-exposure image fusion via generative adversarial networks. IEEE Trans. Image Process..

[48] Chen J., Li X., Luo L. (2020). Infrared and visible image fusion based on target-enhanced multiscale transform decomposition. Inf. Sci..

